# Heterogeneity of cancer-associated fibroblasts in head and neck squamous cell carcinoma: opportunities and challenges

**DOI:** 10.1038/s41420-023-01428-8

**Published:** 2023-04-13

**Authors:** Chen Hu, Yifan Zhang, Chunping Wu, Qiang Huang

**Affiliations:** 1 Department of Otolaryngology, Head and Neck Surgery, Beijing TongRen Hospital, Capital Medical University, 100730 Beijing, China; 2 Department of Otorhinolaryngology, Eye & ENT Hospital, Fudan University, 200031 Shanghai, China

**Keywords:** Head and neck cancer, Cancer microenvironment

## Abstract

Head and neck squamous cell carcinoma (HNSCC) is among the most severe and complex malignant diseases with a high level of heterogeneity and, as a result, a wide range of therapeutic responses, regardless of clinical stage. Tumor progression depends on ongoing co-evolution and cross-talk with the tumor microenvironment (TME). In particular, cancer-associated fibroblasts (CAFs), embedded in the extracellular matrix (ECM), induce tumor growth and survival by interacting with tumor cells. Origin of CAFs is quite varied, and the activation patterns of CAFs are also heterogeneous. Crucially, the heterogeneity of CAFs appears to play a key role in ongoing tumor expansion, including facilitating proliferation, enhancing angiogenesis and invasion, and promoting therapy resistance, through the production of cytokines, chemokines, and other tumor-promotive molecules in the TME. This review describes the various origin and heterogeneous activation mechanisms of CAFs, and biological heterogeneity of CAFs in HNSCC is also included. Moreover, we have highlighted versatility of CAFs heterogeneity in HNSCC progression, and have discussed different tumor-promotive functions of CAFs respectively. In the future, it is a promising strategy for the therapy of HNSCC that specifically targeting tumor-promoting CAF subsets or the tumor-promoting functional targets of CAFs.

## Fact

1. The origins of CAFs are quite varied, and the main sources of CAFs in TME are local NFs. Other sources of CAFs include MSCs, epithelial cells, adipocytes, pericytes and endothelial.
2. CAFs are divided into different subclusters (myCAFs, iCAFs and apCAFs) according to their characteristic marker genes, and these CAFs subclusters showed distinct phenotypes in HNSCC.
3. CAFs play a variety of roles in the progression of HNSCC (proliferation, invasion, migration, angiogenesis, lymphangiogenesis, EMT, and immunosuppression), and CAFs may lead to poor prognosis.
4. It is a promising strategy for the future therapy of HNSCC that specifically targeting tumor-promoting CAF subsets or the tumor-promoting functional targets of CAFs.

## Open questions

1. What are the mechanisms and signaling pathways involved in the activation of CAFs in HNSCC?
2. What are the detailed molecular mechanisms by which the heterogeneous versatility of CAFs is involved in HNSCC progression?
3. CAFs may have complex functions as both tumor-promoting and tumor-suppressing agents, and how to specifically target the tumor-promoting function of CAFs?

## Introduction

The most prevalent head and neck cancer type is squamous cell carcinoma (HNSCC), which develops from the mucosal epithelium of the oral cavity, hypopharynx, and larynx [1]. Despite recent advances in HNSCC therapy, the outcome of advanced patients remains poor [2]. Therefore, creating innovative and effective medicines powered by conceptually transformational basic science is urgent. Only a portion of the cancer development process can be explained by the conventional tumor cell-centric theory of the disease. Therefore, it is crucial to have a thorough understanding of the tumor microenvironment (TME).

Through paracrine, juxtacrine, and autocrine connections, the surrounding TME co-evolves into an active state during HNSCC development, resulting in a dynamic signaling circuitry that aids in the initiation, progression, and resistance to therapy [3]. Cancer-associated fibroblasts (CAFs) play numerous functions in the formation of tumors inside the TME, and related research has shown that CAFs can enhance tumor development in myriads of ways [4–6]. Our knowledge of CAF biology is incredibly dependent on their functional variations based on the heterogeneity of CAFs, various CAF subtypes, and their distinct impacts on tumor behavior. This review summarizes current findings about the significance of CAF heterogeneity in the development of HNSCC and discusses CAF targeting in anticancer therapies.

## Origins of CAFs

The TME has been identified as one of the variables contributing to the growth and invasion of HNSCC [7]. The constantly active fibroblasts known as CAFs, which are found inside or close to the tumor mass, have been linked to having a solid HNSCC modifying impact and playing a significant role in areas such as drug resistance [7–9]. CAFs are spindle-shaped cells and a highly heterogeneous intra-tumoral population of fibroblasts expressing enhanced cellular migration, elevated proangiogenic cytokine signaling, and increased autocrine growth factor-induced signaling [3, 10].

**Fig. 1:**
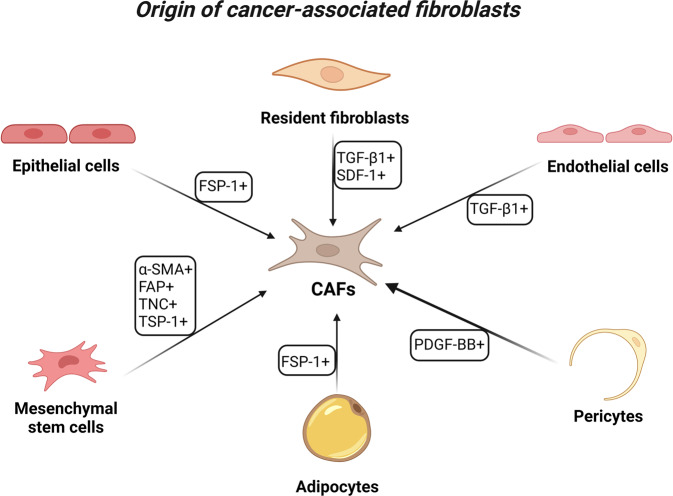
Different origins of cancer-associated fibroblasts (CAFs) in cancer. The origin of CAFs can be quite heterogeneous, and the main sources of CAFs in TME are NFs. Growth factors like TGF-1 and stromal SDF-1 can be secreted by tumor cells to enable the conversion of NFs into CAFs, and CAFs in the tumor stroma do not undergo apoptosis. Other sources of CAFs include direct generation from MSCs, which can migrate to tumor sites in a way akin to fibroblast migration during wound healing. These migratory cells are drawn to cancer and differentiate into CAFs as a result of their attraction to the disease. In their cytoplasm, these CAFs exhibit particular markers such -SMA, FAP, TNC, and TSP-1. Moreover, epithelial cells can undergo EMT to develop into CAFs, and these CAFs maintain the genetic changes made to the parental genome. Due to the expression of mesenchymal lineage-committed marker genes, CAFs also originate from adipocytes. Endothelial and pericyte cells have the ability to transdifferentiate and add to the CAF population. Proliferating endothelial cells can undergo endothelial to mesenchymal conversions to develop CAFs under the effect of TGF-1 produced by cancer. By the influence of PDGF-BB, pericytes are also a source of CAFs. (Created with BioRender.com).

The origin of CAFs can be quite varied (Fig. 1), and the main sources of CAFs in TME are local normal fibroblasts (NFs). Tumor cells secrete growth factors, such as transforming growth factor-beta1 (TGF-β1) and stromal cell-derived factor-1 (SDF1), to facilitate the transformation of NFs into CAFs [11–14]. CAFs are drawn to the tumor site in a process similar to how they are pulled to the site of wound healing [15, 16]. The activated fibroblasts in the tumor matrix do not undergo apoptosis, a typical outcome of activated fibroblasts during typical wound healing. Instead, they constantly interact with tumors; hence, cancers are also known as “wounds that never heal” [17, 18].

There are various other sources of CAFs, and direct production of CAFs from mesenchymal stem cells (MSCs) is possible [19], which also reach tumor sites using processes similar to fibroblast migration during wound healing. There is evidence that these migratory cells are attracted to cancer and undergo differentiation into CAFs. These CAFs express specific markers such as α-smooth muscle actin (α-SMA), fibroblast-activation protein (FAP), tenascin-C (TNC), and thrombospondin-1 (TSP-1) in their cytoplasm [20]. CAFs also arise from adipocytes due to the expression of mesenchymal lineage-committed marker genes [21]. Moreover, CAFs also can arise from epithelial cells via epithelial–mesenchymal transition (EMT), and EMT-derived CAFs retain the genetic alterations in their parental genome [4, 19]. Although EMT-derived CAFs seldom make up the majority of CAFs, several data point to the development of CAF mutations. However, Wang et al. [22] focused on the karyotype variations and biological characteristics between primary cultured CAFs and NFs. According to this model, HEp-2 laryngeal cancer cells could not produce CAFs via EMT during tumor growth. Pericytes and endothelial cells can transdifferentiate and contribute to the population of CAFs. Under the influence of TGF-β1 released by cancer, proliferating endothelial cells can undergo endothelial to mesenchymal transitions to become CAFs [19, 23]. Part of CAFs also derives from pericytes through the influence of platelet-derived growth factor-BB (PDGF-BB) [24]. All these CAF origins are not mutually exclusive and produce a vastly heterogeneous subpopulation of CAFs within individual cancer types. This could be the reason for the reported variations in the identification markers for CAFs.

## Activation of CAFs in cancer

A sea of signaling pathways regulates the activation and reprogramming of CAFs. Shimoda et al. [25] confirmed that complete knockout of tissue inhibitor of metalloproteinases (TIMP) is sufficient to obtain the hallmark CAFs function. Similarly, loss of expression of the tumor suppressor p85α results in fibroblasts in the stroma that can acquire characteristics of CAFs [26]. Failure of the Notch effector protein CSL [27] or upregulation of the proto-oncogene YAP1 [28] in fibroblasts is sufficient to activate CAFs and promote tumorigenesis. However, it is still unclear which epithelial-stromal cell or stromal-stromal cell cross-talk and other mechanisms activate fibroblasts in fibroblasts harboring mutations or alterations in the expression of these genes. Numerous, and possibly separate, external cues that activate fibroblasts in the TME are present in various tumor types. The anti-invasive or pro-invasive effects of CAFs may be activated by a particular combination of growth factors and cytokines that are unique to each tumor type. In this regard, the contractile and invasive characteristics of CAFs have been linked to the leukemia inhibitory factor (LIF) [29, 30]. Increasing WNT7A synthesis by invasive cancer cells in breast cancer may improve TGF-receptor signaling related to the invasive characteristics of CAFs [31]. Vitamin D receptor activation may decrease the CAFs’ tumor-promoting secretome in pancreatic ductal adenocarcinoma (PDAC) [32]. Therefore, most current studies are more inclined to believe that various regulators released by tumor cells activate quiescent fibroblasts or other stromal target cells and reprogram their activation into irreversible CAFs [33, 34].

In a host of studies on HNSCC, NFs are fibroblasts extracted from normal tissue distant from cancer. They can be activated in vitro or in vivo, and activated NFs are found to possess abilities that facilitate cancer [7, 35–37]. Kim et al. found that this process can be induced via co-culturing NFs with oral squamous carcinoma cells (OSCCs) in vitro [38, 39]. Other studies have also reflected a familiar phenomenon in the treatment with TNF-α [40] and TGF-β1 [41–43]. TGF-β1 is regarded as a traditional activator of the phenotype known as myofibroblastic CAFs, which is distinguished by the upregulation of α-SMA. HNSCC cells reportedly interact with skin dermal fibroblasts [44, 45], according to investigations that administered either TGF-β1 or LIF, two distinct activation activators, which led to the same epigenetically controlled CAFs phenotype [45]. Notably, TGF-β1 and fibroblast growth factor (FGF) cause opposing regulation of CAF effector genes, demonstrating that various activation approaches may result in various CAF phenotypes, with consequent diverse effects on the growth of cancer [45]. However, in contrast to an experimental context, the interactions that result in the in vivo activity of fibroblasts are more complex. In addition, extracellular vesicles (EVs) are substances with lipid bilayers that most cells release into their environment [46, 47], and EVs have been thought to be a way of expelling toxic or unnecessary internal substances from cells, and they are also significant facilitators of cell-to-cell communication [48]. EVs can transport a variety of cargoes that can send messages to destination cells to cause metabolic responses, they can also translocate into the cytoplasm of target cells, become active, and then regulate how secretory cells interact with the extracellular matrix there [49]. The stromal target cells of tumor cell-derived EVs are mainly fibroblasts, which dynamically regulate each other in the tumor microenvironment, diverse HNSCC-derived EVs cargoes, such as nucleic acids, signaling proteins, and metabolites, contribute to the activation of CAFs (Table 1).

**Table 1:** Fibroblast activation and reprogramming mediated by HNSCC-derived EVs.

Cancer type	EVs-secreted content	Stromal target cells	Mechanism	References
Head and neck squamous cell carcinoma	miR-192/215	Human foetal lung fibroblasts (MRC-5cells)	Targeting regulating Caveolin-1	[134]
Oral squamous cell carcinoma	LncRNA CAFs	Oral fibroblasts	Regulating IL-33	[135]
Head and neck squamous cell carcinoma	TGFβ1	Head and neck fibroblasts	Regulating Fibronectin	[43]
Nasopharyngeal carcinoma	LMP1	Nasopharyngeal fibroblasts	NF-κB p65 signaling pathway	[103]

## Biological heterogeneity of CAFs in HNSCC

To isolate or identify CAFs, a variety of intracellular, extracellular, and cell surface proteins have been utilized. However, there is no common marker for CAF research. For instance, upregulation of α-SMA has previously been thought to be a distinguishing factor between activated CAFs and NFs [50]. However, a recent study by Michael Bartoschek et al. demonstrated that the existence of reduced α-SMA CAFs highlights the necessity of taking into account a number of characteristics when analyzing CAFs activation [51–53]. Furthermore, pericytes, lymphatic endothelial cells, and fibroblastic reticular cells, in that order, show significant levels of some proteins that are extensively expressed among CAFs, including α-SMA, podoplanin (PDPN), and FAP [4]. A selection of the reported markers of HNSCC CAFs is summarized in Table 2.

**Table 2:** Markers and signature genes of HNSCC CAFs.

CAFs markers/ signature genes	Description of markers/ genes	Expression level in HNSCC CAFs	Biological functions/ notes	References
Vimentin	Type III intermediate filament protein	Upregulated	Promoting tumor EMT/ poor outcome	[70]
α-SMA	Actin isoform	Upregulated	Related with CAFs activation/ poor outcome	[136]
FSP1	Calcium-binding protein containing 2 EF-hand calcium-binding motifs	Upregulated	Identifying CAFs in vitro	[70]
FAP	Membrane-bound gelatinase	Upregulated	Poor outcome	[60, 136]
PDGFRα	Protein tyrosine kinase receptor	Upregulated	Mediating mesenchymal stromal cell chemotaxis to tumor	[137]
PDGFRβ	Protein tyrosine kinase receptor	Upregulated	Signing stromal activation in tumor	[60, 138]
Caveolin-1	Scaffolding protein within caveolar membranes	Upregulated	Inhibited the TGF-β/SMAD signaling and promoting CAFs activation	[134, 139]
Periostin	Secreted extracellular matrix protein, a ligand for α-V/β-3 and α-V/β-5 integrins	Upregulated	Promoting tumor stemness/ poor outcome	[60, 85]
Podoplanin	Mucin-type protein, heavily O-glycosylated glycoprotein	There is no relationship directly	Promoting lymph nodes metastasis/ poor outcome	[140]
CD44	Non-kinase transmembrane glycoprotein	Upregulated	Mediating immunosuppression/ poor outcome	[141]
AKT3	A serine/ threonine protein kinas that modulates various cellular responses via the PI3K-AKT pathway	Upregulated	Mediating immunosuppression and promoting myofibroblastic phenotype/ poor outcome	[60]
COX-2	An inducible enzyme responsible for the production of prostaglandins at sites of inflammation and wound‐healing.	Upregulated	Promoting tumor migration, angiogenesis and invasiveness/ poor outcome	[95, 125]

The roles of CAFs are as varied as their identification markers, and they have dynamically heterogeneous impacts on cancer at various stages [54–56].According recent research, CAFs were divided into “myofibroblastic CAFs” (myCAFs), “inflammatory CAFs” (iCAFs)and “antigen-presenting CAFs” (apCAFs) according to their characteristic marker genes, and these CAFs subclusters showed distinct phenotypes enriched in myofibroblast function, ECM remodeling and antigen-presenting function respectively [57–59]. Among them, myCAFs are characterized by myofibroblasts with high α-SMA expression and enrichment in smooth muscle contraction [57], and ECM-receptor interaction, vascular smooth muscle contraction, and focal adhesion are enriched in myCAFs [59]. Related study found that CAFs in HNSCC mainly comprise myCAFs that play key roles in HNSCC progression, including matrix remodeling, the production of growth factors and cytokines, and metabolic effects [60]. CXCL12 is a vital chemokine, which can promote cancer cells proliferation, angiogenesis, and metastasis [61], and Wang et al. found that CXCL12 is mainly secreted by iCAFs in OSCC [59]. That means iCAFs may facilitate HNSCC progression via some mechanisms. Recent studies have revealed a novel apCAFs in pancreatic ductal adenocarcinoma [62], it was observed that apCAFs were capable of expressing genes associated with the major histocompatibility complex (MHC) class II family. Further evidence that apCAFs might deliver antigens to T cells came from in vivo testing, the expression of costimulatory genes, which are necessary for T cell proliferation, was low in the apCAFs [30]. This suggested that apCAFs could be involved in TME immunosuppression. Zhang et al. found that Cluster 4 expressed high levels of MHC class II family, which was characterized as antigen-presenting CAFs, and that revealed that perhaps apCAFs also exist in HNSCC tissue [57].

In addition, despite the overwhelming theories that CAFs are positive regulators of cancer, however, recent research has shown that some CAFs subtypes can inhibit tumor development through certain mechanisms as well [4, 19]. CAFs have been defined as C1 and C2 types according to low and high α-SMA expression in OSCCs, respectively, and C1-type CAFs suppress the self-renewal of oral stem-like cancer cells (SLCCs) [63]. And that may provide new strategy for anti-cancer therapy of HNSCC in the future.

## Versatility of CAFs heterogeneity in HNSCC progression

In human samples, spindle-shaped stromal cells that express the myofibroblastic marker α-SMA are often used to identify CAFs in HNSCC [64, 65]; other markers are less frequently co-expressed [66, 67]. There may be a subgroup of non-myofibroblastic CAFs, whose functional importance remains unclear, given that positivity for α-SMA is very varied [64, 65, 67]. Chemotherapy could also result in upregulation of α-SMA [68]. Based on the myofibroblastic phenotype, the clinical and prognostic characteristics of CAFs have been discovered. Vered et al. found that worse clinical parameters [69, 70] and terrible outcome [67, 71–73] are likely linked with high numbers of CAFs. CAF-derived products grant tumors different promoting abilities [63, 74, 75], and the possible subtypes of CAFs must be considered when assessing those differences [63, 74, 75]. For example, NFs were induced by HPV-negative oropharyngeal cells to release hepatocyte growth factor (HGF) and IL-6, which might encourage tumor cell formation by activating c-met and STAT3. While Oropharyngeal cells that were HPV-positive did not work [76]. A selection of the reported markers and genes of CAFs is summarized in Table 2.

**Fig. 2:**
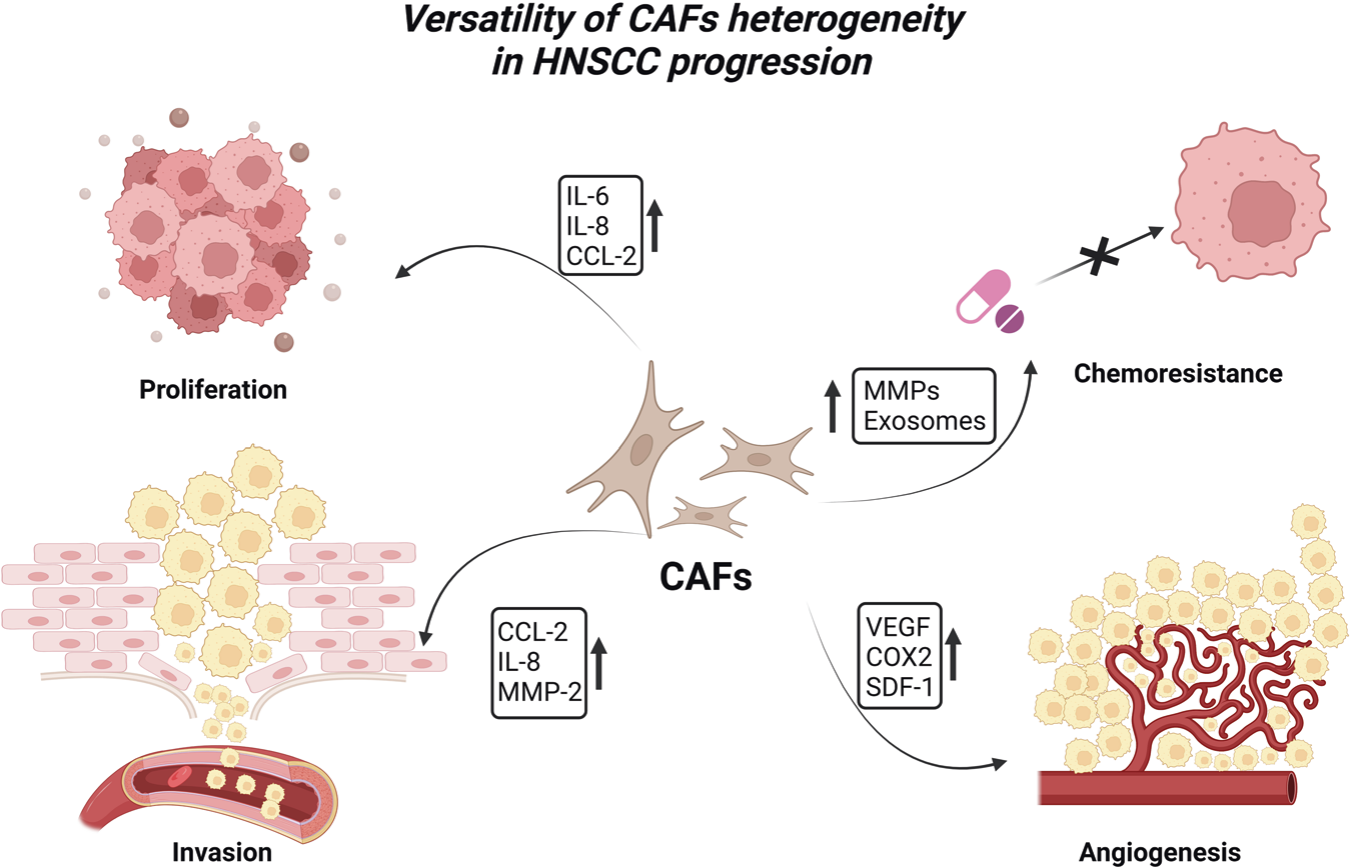
Versatility of cancer-associated fibroblasts (CAFs) heterogeneity in HNSCC progression. By the synthesis of cytokines, chemokines, and the extracellular matrix (ECM) in the TME, CAFs perform a range of roles in carcinogenesis, including proliferation, chemoresistance, invasion, and angiogenesis. As epithelial cells undergo the transformation to become CAFs during the premalignant stage, it is believed that the primary function of CAFs in the development of cancer is to stimulate the proliferation of cancer cells. In the coculture of CAFs/NFs-OCCs, CAFs but not NFs facilitated OCC cell proliferation and invasion, and MMP-2 derived from senescent CAFs induced keratinocyte dis-cohesion and epithelial invasion into collagen gels in a TGF-β-dependent manner. In addition, CAFs can cause angiogenesis in HNSCC by producing PGE2 through COX-2, and VEGF and SDF-1 are required for CAFs to stimulate neoangiogenesis in the tissue of NPC. Moreover, by secreting exosomes to the tumor, CAFs can confer HNSCC therapy resistance, such as chemoresistance. (Created with BioRender.com).

The procedure for promoting tumor growth involves several channels and signals. Numerous studies have demonstrated that CAFs play a variety of roles in carcinogenesis, including proliferation, invasion, and metastasis, through the production of cytokines, chemokines, and the extracellular matrix (ECM) in the TME [4, 19, 70, 77–79] (Fig. 2).

### CAFs contribute to proliferation and stemness in cancer

The primary role of CAFs in carcinogenesis is assumed to be related to the process of epithelial cells transforming into CAFs during the premalignant stage. This property of CAFs is thought to be the stimulation of cancer expansion [80]. Simona et al. demonstrated that OSCC CAFs-derived microfibrillar-associated protein five could increase phosphorylation of PDK1 and Akt and decrease cRAF and PTEN to induce tumor proliferation [81]. Qin et al. found that HNSCC CAFs-derived IL-6 can promote tumor proliferation by regulating osteopontin expression in a STAT3 dependent-way [82]. In a related study [83], CAFs–OSCC coculture creates a favorable cytokine-rich microenvironment. CAFs produce high chemokine ligand 2 (CCL-2) and promote oral cancer cells (OCCs) proliferation, migration, invasion, and tumor growth.

CAFs can also maintain cancer cell stemness or even encourage the reacquisition of stem-like features via producing insulin-like growth factor (IGF)-II [84]. CAFs play a crucial function in controlling the TME and promoting cancer development because they have a variety of effects on cancer stemness and proliferation. According to a related study [85], Binbin Yu et al. demonstrated a notable relationship among PTK7, Wnt/β-catenin pathway, and aggressive clinicopathologic features in HNSCC. Further, a co-immunoprecipitation (co-IP) assay confirmed that periostin secreted by CAFs might act as a receptor because it is a potential upstream ligand of PTK7. Further analysis revealed that periostin boosted the tumor stem cell (CSC)-like phenotype through PTK7-Wnt/β-catenin signaling, inducing HNSCC cell migration in vitro. The study also assessed tumor initiation and progression in vivo. The results suggest that the combination of periostin and PTK7 might be a potential prognostic and diagnostic indicator and a promising therapeutic target.

### CAFs promote invasion, migration, and EMT in cancer

Accumulating evidence suggests that CAFs can promote tumor development in other ways, such as invasion and migration, via certain mechanisms [86]. For instance, Li et al. demonstrated that in a coculture of CAFs/NFs-OCCs, CAFs but not NFs promoted OCC cell proliferation and migration [87]. Matrix metalloproteinase (MMP)-2 derived from senescent CAFs-CM induced keratinocyte dis-cohesion and epithelial invasion into collagen gels in a TGF-β-dependent manner. Hassona et al. [88] demonstrated that senescent CAFs from genetically unstable OSCC promote a more aggressive oral cancer phenotype through the production of active MMP-2, disruption of epithelial adhesion, and induction of keratinocyte invasion. According to a study by Qin et al. [89], periostin is overexpressed in HNSCC and linked with the proliferation and spread of tumor cells. Periostin was found to be substantially expressed in CAFs and hardly expressed in NFs according to western blotting, real-time PCR, and semi-quantitative RT-PCR examinations. Therefore, they thought that the primary source of periostin in HNSCC tissues was the cancer stroma, particularly CAFs.

Among the most important ways that CAFs promote carcinogenesis is via EMT. This mechanism gives tumor cells the ability to move about and invade nearby tissues, which leads to the dispersal of tumor cells and the progress of metastatic disease [90]. Upregulation of EMT markers is common in primary and metastatic cancer, as shown by immunohistochemical analyses of SCC, indicating that there is a strong connection between CAFs and cancer-promoting functions, such as EMT induction and metastasis [91]. Furthermore, TNFa facilitates the myofibroblast differentiation of NFs to CAFs. CAF-derived SDF1 can separately serve as an independent factor to enhance metastasis, EMT, and angiogenesis of tongue cancer [40].

### CAFs promote angiogenesis and lymphangiogenesis

Prior research concentrated on the TME’s vascularization and immunological response because of their potential as therapeutic targets [92, 93]. Many models have supported the contribution of CAFs to tumor vascularization. For instance, PDGFR-expressing CAFs promote cancer cell growth and angiogenesis in a model of HPV cervical carcinogenesis. Cancer cell-derived PDGF promotes FGF2 production, which produces a proangiogenic impact. This effect is eliminated by pharmaceutical therapy that blocks stromal PDGFR signaling [94]. CAFs are reported to induce angiogenesis in HNSCC via COX-2-mediated production of prostaglandin E2 (PGE2) [95, 96]. In addition to increasing invasion and vein formation, oral CAFs have been demonstrated to induce tumor vascularization [40]. In nasopharyngeal carcinoma (NPC), Wang et al. found that CAFs are closely linked to angiogenesis. They showed that fibroblasts from NPC tissues have elevated levels of α-SMA expression, and that the stroma of NPC tissues also included elevated endothelial progenitor cells, which promote neoangiogenesis in a VEGF- and SDF-1 dependent way [97]. Further, they demonstrated that fibroblasts from NPC tissues have much elevated levels of α-SMA expression, and that the stroma of NPC tissues also included elevated endothelial progenitor cells, which promote angiogenesis in a VEGF- and SDF-1-dependent way. In the TME, lymphatic vessel remodeling facilitates cancer progression and metastasis [98]. To efficiently study the role of CAFs in lymphatic vessel conditioning in the context of HNSCC, Karina et al. built lymphatic organotypic coculture models using HNSCC-derived CAFs [9]. They found that HNSCC-derived CAFs can induce lymphatic vessel sprouting to alter lymphatic vessel permeability and cause gene expression changes in lymphatic vessels.

### CAFs regulate the immune systems

Cytokines released by CAFs also control the polarization and recruitment of immune cells. Hideyuki et al. [99] found that compared with control cells, CAF-educated cells suppressed T cell proliferation more strongly, and the neutralization of TGF-β, IL-10, or arginase I significantly restored T cell proliferation. According to their findings, CAFs regulate the tumor immunosuppressive microenvironment in OSCCs by fostering the protumoral phenotype of tumor-associated macrophages (TAMs). Furthermore, this potential role of CAFs in regulating immune cells and vascular demonstrates the complications of CAFs and their potential application in anticancer therapies. In the follow-up study, the team demonstrated that AKT3 expression in CAFs promoted immunosuppression in HNSCC and related to poor prognosis [60]. T cells cocultured with AKT3 knockdown CAFs proliferated more than control cells. In addition, AKT3 knockdown CAFs showed reduced proliferation and migration compared with controls, and overall survival (OS) was significantly shorter in patients with AKT3-positive CAFs.

Furthermore, in order to compared the effects between CAFs and NFs on T cell proliferation in HNSCC, Hideyuki et al. co-cultured CAFs and NFs with fluorescent cell staining dye -labeled T cells, and found that the suppressor activity of CAFs was greater than that of NFs [100]. In addition, the suppression of T cell proliferation by the culture supernatant from CAFs was higher than that from NFs. And they demonstrated that the supernatant from CAFs induced T cells apoptosis and regulatory T cells. Those results elucidated that CAFs collaborated with tumor cells in the TME to establish an immunosuppressive network that facilitated tumor evasion from immunological destruction. Moreover, Huang et al. [101] found that there is a negative correlation between WNT2+ CAFs and active CD8+ T cells was detected in OSCC, and their subsequent mechanistic analyses demonstrated that CAFs-derived WNT2 inhibit the dendritic cell (DC) -mediated anticancer T-cell response via the SOCS3/p- JAK2/p- STAT3 signalling cascades.

### CAFs induce cancer progression via metabolic changes

Metabolic reprogramming most frequently results from the TME. Tumor cells are well recognized for altering their metabolism to sustain high proliferation rates and survive in adverse conditions with limited oxygen and nutritional deficiencies [102]. A growing body of research indicates that CAFs play a crucial role in regulating tumor metabolism, particularly through dysregulating various metabolic pathways, such as glucose, amino acids, and lipid metabolism. These actions aid in the development, spread, and resistance of tumor cells to treatments [103–106].

Cancer cells perform glycolysis rapidly, even under aerobic conditions, thus increasing glucose uptake and lactate secretion, an effect called the “Warburg effect” or “aerobic glycolysis” [107]. Tumor cells hijack CAFs and reprogram their metabolism; under the impact of cancer cells, CAFs display comparable aerobic glycolysis, which is known as the reverse “Warburg effect” [106]. In contrast to NFs, CAFs significantly switch from aerobic glycolysis to oxidative phosphorylation, and can secrete products such as pyruvate and lactate to meet the metabolic needs of cancer cells. Zhang and colleagues observed that OSCC-derived CAFs exhibited significantly higher integrin beta 2 (ITGB2), and increased expression of ITGB2 in CAFs was associated with a poor prognosis. ITGB2 controls the PI3K/AKT/mTOR pathway, which increases CAFs’ glycolytic activity. Lactate generated by CAFs with ITGB2 overexpression is subsequently absorbed by cancer cells, promoting the growth of OSCCs [108]. NPC has been linked to the lactate shuttle phenomenon [103]. The investigators noted that inhibiting monocarboxylate transporter 4 (MCT4) upregulation in activated CAFs significantly reduced nasopharyngeal carcinoma cell proliferation, invasion, and colony formation. Moreover, Dhruv Kumar et al. [109] found that CAFs-secreted HGF facilitates HNSCC progression: HNSCC cancer cells and CAFs have a metabolic relationship in which CAFs secrete HGF to induce a glycolytic switch in HNSCC cells, and HNSCC cells secrete basic FGF to promote lactate consumption by CAFs.

### CAFs confer cancer therapy resistance

The TME can impose a substantial impact on how well a therapy works since it continuously supplies pro-tumorigenic substances both during and after therapy, reducing the lethal effect on tumor cells [110, 111]. Standard therapies for HNSCC have not changed much over the years, and treatment resistance is still an issue [112]. According to investigations, CAFs promote the survival of HPV-negative HNSCC after cisplatin treatment [7, 113], as well as HPV-positive and negative cells that receive cetuximab [114, 115] and mTOR inhibitor therapy [114]. MMP-1 was shown to be more highly expressed in both the cancer cells and the CAFs when HNSCC were cocultured with them, according to Johansson et al. [115]. Furthermore, the addition of an MMP inhibitor partially eliminated CAF-induced tolerance. The fact that CAFs treated with siRNA directed against MMP-1 continued to shield cancer cells from cetuximab therapy suggests that either several MMPs may work together to promote resistance, or that a different MMP family member underlies the protective effect. Johansson et al. discovered that cetuximab-induced growth suppression was lessened when cancer cells were cocultured with CAFs in a transwell system, indicating that the resistance to treatment was caused by soluble substances produced from CAFs [115]. When HNSCC cell lines and CAFs were cocultured, MMP-1 expression was increased in both the cancer cells and the CAFs. Furthermore, the addition of an MMP inhibitor partially eliminated CAF-induced resistance. These findings indicate that the unique modification of cetuximab sensitivity is CAF-dependent and imply that blocking MMPs could enhance the benefits of EGFR-targeted treatment.

Furthermore, CAFs can target specific drugs or treatment modalities through EV-mediated tumor therapy resistance, making the results more accurate. Qin et al. [7] showed that HNSSC CAFs are intrinsically resistant to cisplatin, and CAFs can secrete miR-196a to tumor cells through exosomes. Consequently, miR-196a released from exosomes can confer cisplatin resistance to cancer cells by novel binding targets, CDKN1B and ING5, demonstrating that miR-196a could be a potential predictor in HNSCC and may become a potential therapeutic target for cisplatin resistance. Moreover, Peltanova et al. [116] found that CAFs from different patients had different sensitivity for cisplatin, and demonstrated that CAFs could enhance and/or inhibit colony-forming capability and chemoresistance in HNSCC cells via paracrine effects and subsequent changes in gene expression of cancer-associated genes in cancer cells.

There are various immune escape mechanisms in cancer, such as suppression of lymphocyte infiltration into the tumor mass. Ford et al. [117] found that CAF induce immunotherapies resistance via specifically excluding CD8 + T-cells from the tumor mass, and they found that by the inhibition of NOX4, CAF can be precisely targeted to decrease and revert CAF differentiation, inducing CD8+ T-cell infiltration, and restore the sensitivity of CAF-rich tumor to immunotherapy. In the therapy of anticancer, there exist association between CAFs subtypes and immunotherapy resistance: different CAF subtypes express distinct immunosuppressive factors [118]. CAF subtypes were linked to various clinical prognosis, and researchers discovered important molecular pathways that might either trigger or repress cancer growth, or were involved in resistance to antiPD1 or anti-PD-L1 immunotherapy [118]. In addition, Ksenis et al. [119] showed that higher TGF-β pathway activity in CAFs is correlated with HNSCC resistance to cetuximab. These TGF-activated CAFs produce substances that limit cetuximab’s ability to fight tumors, both in vitro and in vivo. Cetuximab’s effectiveness was increased, and HNSCC development was stopped by just blocking TGF-β signaling.

## Anticancer therapy based on targeting CAFs

Cancer cells, immune cells, and other stromal cells, such as CAFs, constantly interact with one another. Over the past 10 years, anticancer treatments that target CAFs have attracted a lot of attention. FAP is a kind of serine protease that can regulate the physiological function of myofibroblasts and is frequently utilized in studying the activation of CAFs [120]. Targeting FAP in preclinical animals has demonstrated antitumor effectiveness. In mice, the reduction of cells that produce FAP results in a therapeutic vaccine against cancers with TNF and IFN [121]. A related study reported that CAFs, but not HNSCC cell lines, secrete HGF [122], both HGF and c-Met levels are increased in HNSCC compared with normal mucosa, and that HGF acts in a paracrine manner to facilitate HNSCC cell proliferation and invasion [123]. Dhruv Kumar et al. [124] have demonstrated for the first time that the HGF-targeted antibody ficlatuzumab inhibits CAFs-facilitated HNSCC cell migration, invasion, and proliferation. Li et al. [87] demonstrated the dysregulation of miR-124 in oral CAFs and OSCCs compared with matched NFs, and showed that restoring miR-124 expression by lentiviral infection or formulated miR-124 injection inhibited cancer growth in vivo, indicating miR-124 rescue may become a potential therapeutic application in OSCC in the future. Furthermore, Zhu et al. discovered that COX-2 elevates tumor necrosis factor-α expression in CAFs to promote NPC cell migration and invasiveness [125]. Importantly, they discovered that inhibiting COX-2 and prostaglandin E2, a downstream product of COX-2, as well as knocking down COX-2 expression in vivo, induced invasion and metastasis in vitro, indicating that the identification of this COX-2-mediated axis may present odds for targeting CAFs in advanced NPC. In addition, Hideyuki et al. [60] elucidated the function of AKT3 of CAFs in mediating immunosuppression and promoting myCAFs, and OS was significantly shorter in patients with AKT3-positive CAFs. T cells that were cocultured with conditioned medium from AKT3 knockdown CAFs proliferated more than control cells; AKT3 knockdown CAFs showed a reduced proliferation and migration compared with controls. These findings suggest the viability of a new therapeutic modality that targets CAFs in HNSCC.

It is a kind of intriguing strategy: focusing on cytokines and other elements that are connected to CAF biology [126]. The JAK2/STAT3 pathways are intriguing targets for CAF activation, while the constitutive activation of STAT3 by numerous signaling pathways and other variables has made the creation of STAT3 inhibitors challenging over time [127]. Notably, Kasembeli et.al developed TTI-101, a competitive inhibitor of STAT3 that has demonstrated target engagement, little toxicity, and evidence of clinical benefit in a Phase I study in patients with solid tumors (NCT03195699). And they found TTI-101 didn’t have an impact on mitochondrial activity, STAT3 aggregation, chemical modification, or neuropathic pain in subsequent research [128]. Furthermore, both in vivo and in vitro JAK2 inhibitors have demonstrated to reduce HNSCC proliferation in previous study [129]; Ruxolitinib, a JAK1/2 inhibitor approved for myelofibrosis, has being evaluated in operable HNSCC (NCT03153982). In addition, FAP is expressed scarcely in common condition, and its high and restricted expression on reactive stroma in TME makes FAP a promising target for anticancer therapy. FAP-IL2v is an immunocytokine comprising an antibody against FAP and an IL-2 variant, and FAP-IL2v demonstrated ability of targeting tumor according to imaging studies results [130]. Moreover, in vitro and vivo, FAP-IL2v significantly promoted the activity of therapeutic antibodies that mediate antibody-dependent or T cell-dependent cellular cytotoxicity (TDCC) and of programmed death-ligand 1 (PD-L1) checkpoint inhibition. In the clinical trial about combination therapy of FAP- IL2v with trastuzumab and cetuximab (NCT02627274), rapid expansion of CD8 cells and NK cells, but not Tregs, was observed in treated patients. The result indicated that FAP-IL2v is a promising immunocytokine that potentiates the efficacy of anticancer immunotherapies.

## Conclusion and perspective

Treatment options for HNSCC are increasingly abundant, but the prognosis of patients is still unsatisfactory. CAFs play an essential role in HNSCC progression, and their functional differences are based on the heterogeneity of CAFs. Some studies have suggested that CAFs or fibroblasts have detrimental impacts on tumors that include a number of factors, which is contrary to the predominant idea that CAFs always facilitate tumor [131–133]. Oral CAF subtypes with a lower score for α-SMA (C1-type CAFs) were demonstrated to be more likely to induce cell proliferation but suppress the self-renewal growth of oral SLCCs, and Ankit et al. found the decisive role of bone morphogenetic protein 4 in C1-type CAFs-mediated suppression of self-renewal of oral SLCCs [63].

However, the heterogeneity of CAFs has not been studied extensively in HNSCC, and the mechanism of their impact on tumor progression remains unclear. The actual biological functions of different groups of CAFs in tumorigenesis still need to be validated using various tumor models and clinical data. Given that CAFs may have complex functions as both tumor-promoting and tumor-suppressing agents, potential therapeutic strategies targeting CAFs should specifically target tumor-promoting CAF subsets or the tumor-promoting functional targets of CAFs.

## Data Availability

All data included in this review are available upon request by contact with the corresponding author.
